# Associations over the COVID-19 pandemic period and the mental health and substance use of youth not in employment, education or training in Ontario, Canada: a longitudinal, cohort study

**DOI:** 10.1186/s13034-023-00653-4

**Published:** 2023-09-07

**Authors:** Meaghen Quinlan-Davidson, Di Shan, Darren Courtney, Skye Barbic, Kristin Cleverley, Lisa D. Hawke, Clement Ma, Matthew Prebeg, Jacqueline Relihan, Peter Szatmari, J. L. Henderson

**Affiliations:** 1 Centre for Addiction and Mental Health, Toronto, ON Canada; 2 Department of Psychiatry, University of Toronto, Toronto, ON Canada; 3 Faculty of Medicine, Department of Occupational Science and Occupational Therapy, the University of British Columbia, Vancouver, Canada; 4 Foundry British Columbia, Vancouver, BC Canada; 5 Lawrence S Bloomberg Faculty of Nursing, University of Toronto, Toronto, ON Canada; 6 Division of Biostatistics, Dalla Lana School of Public Health, University of Toronto, Toronto, ON Canada; 7 The Hospital for Sick Children, Toronto, ON Canada

**Keywords:** Youth not in education, employment or training, COVID-19, Youth mental health and substance use

## Abstract

**Background:**

The economic shutdown and school closures associated with the COVID-19 pandemic have negatively influenced many young people’s educational and training opportunities, leading to an increase in youth not in education, employment, or training (NEET) globally and in Canada. NEET youth have a greater vulnerability to mental health and substance use problems, compared to their counterparts who are in school and/or employed. There is limited evidence on the association between COVID-19 and NEET youth. The objectives of this exploratory study included investigating: longitudinal associations between the COVID-19 pandemic and the mental health and substance use (MHSU) of NEET youth; and MHSU among subgroups of NEET and non-NEET youth.

**Methods:**

618 youth (14–28 years old) participated in this longitudinal, cohort study. Youth were recruited from four pre-existing studies at the Centre for Addiction and Mental Health. Data on MHSU were collected across 11 time points during the COVID-19 pandemic (April 2020-August 2022). MHSU were measured using the CoRonavIruS Health Impact Survey Youth Self-Report, the Global Appraisal of Individual Needs Short Screener, and the PTSD Checklist for DSM-5. Linear Mixed Models and Generalized Estimating Equations were used to analyze associations of NEET status and time on mental health and substance use. Exploratory analyses were conducted to investigate interactions between sociodemographic characteristics and NEET status and time.

**Results:**

At baseline, NEET youth were significantly more likely to screen positive for an internalizing disorder compared to non-NEET youth (OR = 1.92; 95%CI=[1.26–2.91] *p* = 0.002). No significant differences were found between youth with, and without, NEET in MHSU symptoms across the study time frame. Youth who had significantly higher odds of screening positive for an internalizing disorder included younger youth (OR = 1.06, 95%CI=[1.00-1.11]); youth who identify as Trans, non-binary or gender diverse (OR = 8.33, 95%CI=[4.17–16.17]); and those living in urban areas (OR = 1.35, 95%CI=[1.03–1.76]), compared to their counterparts. Youth who identify as White had significantly higher odds of screening positive for substance use problems (OR = 2.38, 95%CI=[1.72–3.23]) compared to racialized youth.

**Conclusions:**

Our findings indicate that sociodemographic factors such as age, gender identity, ethnicity and area of residence impacted youth MHSU symptoms over the course of the study and during the pandemic. Overall, NEET status was not consistently associated with MHSU symptoms over and above these factors. The study contributes to evidence on MHSU symptoms of NEET youth.

**Supplementary Information:**

The online version contains supplementary material available at 10.1186/s13034-023-00653-4.

## Background

The spread of the Coronavirus disease 2019 (COVID-19) led to an economic shutdown and school closures in most countries globally, initially resulting in increased unemployment rates, learning losses, and early school leaving [1]. The impact the pandemic has had on the labour market, educational and training opportunities has been particularly profound on youth, with many forced to abandon school, work, technical and vocational programs [2]. At least temporarily, these disruptions have adversely affected youth’s opportunity to gain skills and knowledge through school, early job experience and training to prepare them for the labour market, during a stage of life when investing in school and job experience is foundational for the future [2]. These lost learning and training opportunities are projected to have an enduring impact on youth, making transitions to the labour market even more challenging for this population group in the years to come [3, 4]. In fact, compared to older age groups, young people (15–24 years of age) are experiencing slower labour market recovery [3, 5], resulting in increased rates of youth not in education, employment or training (NEET) [6]. NEET youth (15–24 years) experience difficult transitions from school to work and face challenges accessing the labour market [7], including temporary disengagement from school, limited job skills and experience, and poor labour market conditions, such as informal and short-term job positions [8].

In Canada, there was an increase in NEET youth over the course of the COVID-19 pandemic, rising from approximately 11% of youth (15–29 years) in 2019 to 14% by 2021 [9]. Current estimates suggest that the percentage of NEET youth has decreased back to 2019 levels at 11% [10]. Similar results were shown among OECD countries, with the proportion of NEET youth (15–29 years) increasing from 13% to 2019 to 15% by 2021. Prior research in Canada has showed that NEET status is common among youth from lower-income and lower education households, contributing to greater vulnerability and social exclusion [11]. NEET status has also been associated with identifying as non-White and older youth age [11, 12]. Studies on sex and gender have been mixed: some studies show NEET status as more common among youth who identify as male [11], while other studies show it as more common among youth who identify as females [12].

NEET youth are more vulnerable to worse mental health symptoms and problematic substance use compared to non-NEET youth [11, 13–15]. In a recent systematic review and meta-analysis, NEET status was associated with symptoms of behavioural and mood disorders, suicidal ideation, psychiatric disorders, and substance use problems among youth [15]. The authors acknowledged, however, that the evidence base was limited, with heterogeneous results [15]. It has been posited that NEET status is negatively associated with MHSU through the following mechanisms: (i) prolonged low self-esteem, including feelings of hopelessness, shame and social exclusion; stigma; and stress attributed to not being in school or having a job [16, 17]; (ii) prolonged stress associated with financial insecurity and hardship; and (iii) prolonged stress associated with searching for jobs and work capability testing [18]. Further research, however, is needed to explore these mechanisms due to the limited evidence available on the topic.

There is some evidence showing a bidirectional relationship between MHSU and NEET status, [8, 15, 19]. Indeed, MHSU problems are associated with negative short- and long-term social and economic factors, including dropping out of school, homelessness, social isolation, incarceration, chronic unemployment, and high economic costs for families, communities and social systems [20–25]. Greater longitudinal research, however, on these mechanisms is warranted.

To mitigate some of the economic and financial impacts experienced by Canadians during the COVID-19 pandemic, the Government of Canada implemented pandemic relief programs [26]. These programs included, among others, the Canada Emergency Response Benefit (CERB) and a top-up to the Goods and Services Tax (GST)/Harmonized Sales Tax (HST). CERB was implemented between March 2020 to September 2020 and provided $500 of taxable income per week for a maximum of 28 weeks [27]. The GST/HST top-up was an additional payment double the annual credit amount to individuals and families with low and modest incomes to offset the costs of these taxes [28]. Focus group research by the Canadian Centre for Policy Alternatives showed that the CERB helped alleviate financial stress and anxiety. The study was conducted among individuals 18 + years of age across British Columbia, Alberta, Saskatchewan, Manitoba, Ontario, Quebec, and the Atlantic region [29].

The various public health strategies implemented in Canada during the COVID-19 pandemic [30], such as restricted movement and physical isolation, and extended disruptions to school, work, and training may be adversely associated with the mental health and wellbeing of youth. Findings, however, have been mixed. Some studies during the pandemic showed decreased hospitalizations due to self-harm among youth [31] and no changes in anxiety symptoms [32, 33]. Other studies have showed that youth reported increased depression, anxiety, post-traumatic stress disorder (PTSD), loneliness, helplessness and substance use during the pandemic [34–42]. These effects could be exacerbated among NEET youth, who face greater social exclusion, compared to non-NEET youth. However, there is a dearth of information about correlates between pandemics and the MHSU of NEET youth [43]. Of the limited research that is available, a global survey among young people (18–29 years) between April and May 2020 showed that 22.6% and 21.9% of young people who stopped working and experienced delayed education, respectively, experienced probable anxiety or depression, compared to 13.5% and 10.7% of those who continued to work and experienced no change in learning [44]. This survey was cross-sectional in nature, conducted at the beginning of the pandemic, and primarily targeted students and young workers. Further, it did not investigate longitudinal associations between the pandemic and youth MHSU.

The lack of evidence on the pandemic’s association with NEET youth limits our ability to meet the MHSU needs of these youth during this critical time and represents a knowledge gap in the literature. As such, the aim of the current exploratory study was to investigate longitudinal associations between the COVID-19 pandemic and the MHSU of NEET youth over the course of two years. We also investigated whether subgroups of NEET youth had a greater vulnerability to MHSU problems over time. Using this approach to identify associations between the pandemic and NEET MHSU will help tailor investments, strategies, and approaches to reach these vulnerable youth and meet their MHSU needs.

## Methods

### Participants and recruitment

**Fig. 1:**
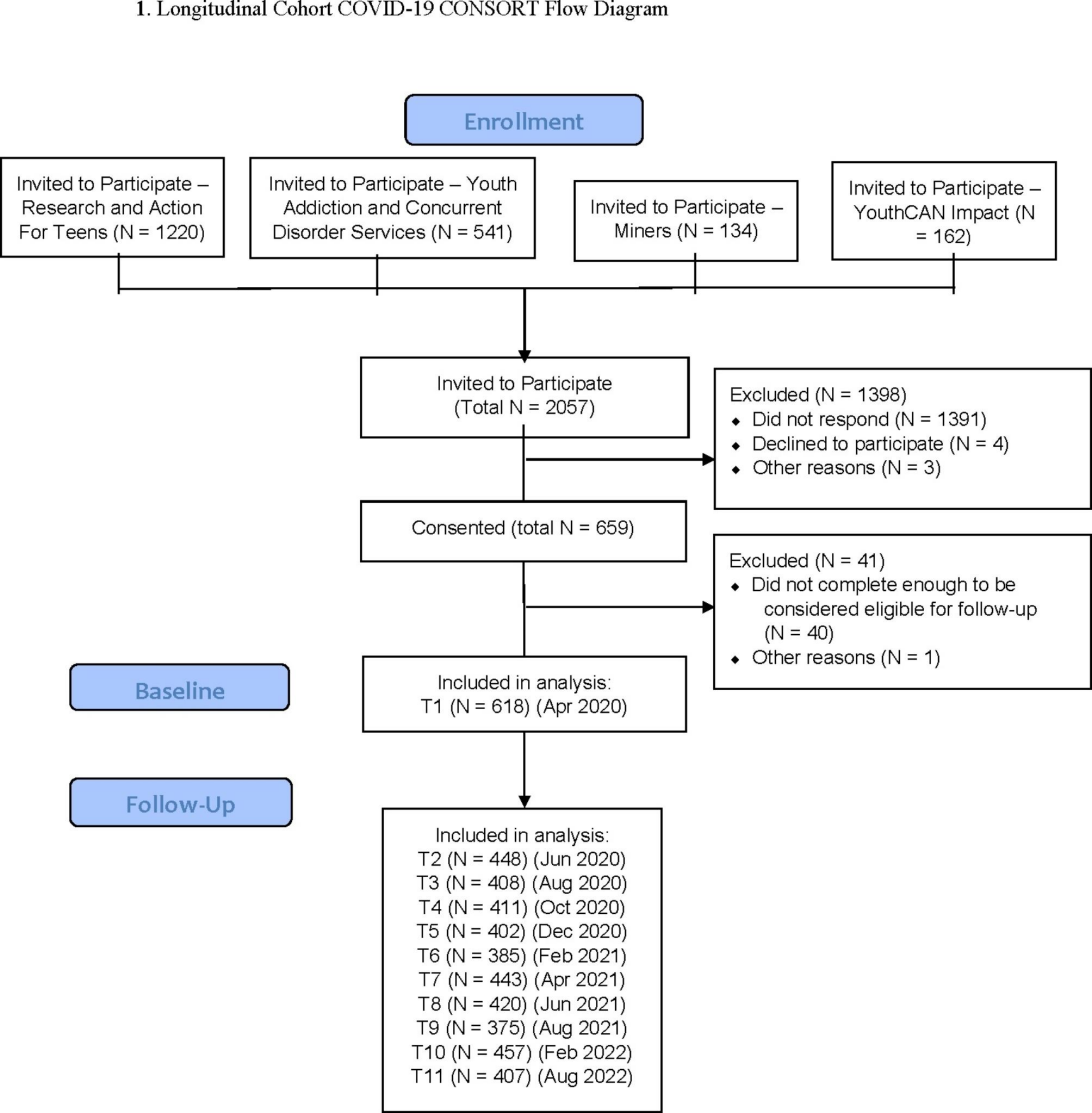
Longitudinal Cohort COVID-19 CONSORT Flow Diagram

This longitudinal cohort study consisted of 618 youth (14–28 years of age), recruited from four pre-existing (three clinical and one non-clinical) CAMH-based studies in Ontario, Canada (Fig. 1) [45–47]. These studies included: (i) n = 71 service-seeking youth from the YouthCan IMPACT study, a randomized controlled trial testing hospital based care to community-based care in youth seeking help with mental health concerns in Toronto [45]; (ii) n = 137 service-seeking youth recruited from Youth Addictions and Concurrent Disorders Services study at CAMH [47]; (iii) n = 67 service-seeking youth from a longitudinal study examining early identification of psychosis spectrum symptoms [48] (iv) n = 343 non-service seeking youth recruited from the Research and Action for Teens study, a community-based longitudinal cohort study [46]. Demographic factors were not accounted for in participant recruitment. Participants from these studies consented to be contacted for future research and were contacted via email for the current study. The study was approved by the Centre for Addiction and Mental Health’s (CAMH) Research Ethics Board, in Toronto, Canada.

**Fig. 2:**
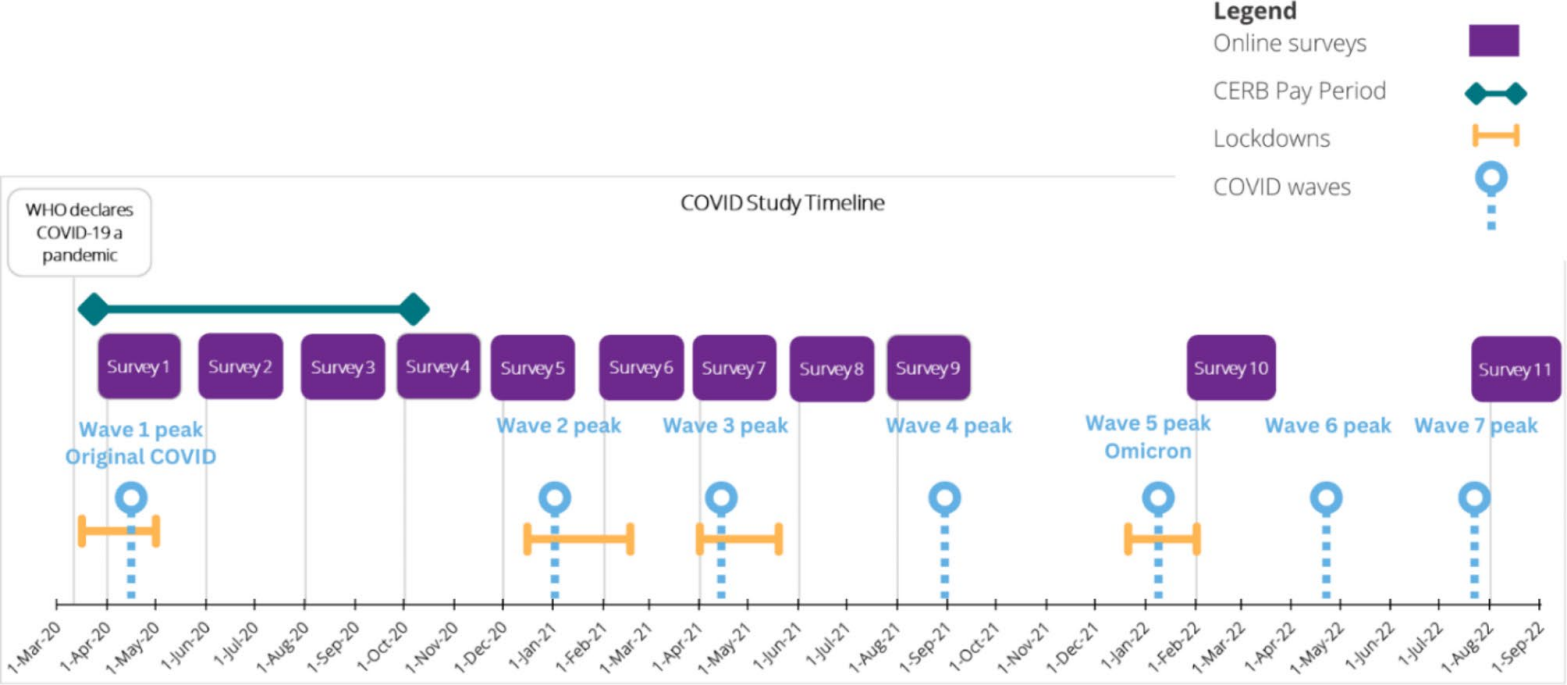
COVID-19 Study Timeline with Survey Time Points, CERB Pay Period, Public Health Measures and COVID waves [54]

*Sources*: Public Health Ontario. (2022). Ontario COVID-19 Data Tool. Toronto, Public Health Ontario; Government of Canada (2022). “Canada Emergency Response Benefit (CERB).“ https://www.canada.ca/en/services/benefits/ei/cerb-application.html; K, N. (2020). A timeline of COVID-19 in Ontario. Global News Toronto, Global News; Government of Ontario (2021). Enhanced Safety Measures in Place as In-Person Learning Resumes Across Ontario: Provincial Medical Officials are Confident Students can Return to Class Safely. Toronto, Government of Ontario; Government of Ontario (2021). Ontario Releases Three-Step Roadmap to Safely Reopen the Province: Province Safely Reopening Outdoor Recreational Amenities Prior to End of Stay-at-Home Order. Toronto, Government of Ontario; Government of Ontario (2021). Ontario Further Strengthening Response to Omicron: Additional measures to slow the spread as province accelerates booster dose rollout. Toronto, Government of Ontario; Canada, G. o. (2022). Ontario Outlines Steps to Cautiously and Gradually Ease Public Health Measures: Time-limited Measures to Blunt Spread of Omicron Protecting Hospital and Health Care Capacity. Toronto, Government of Ontario.

The study included 11 time points (April 2020–August 2022) (Figs. 1 and 2). Data from the first time point (T1) were collected in April 2020, with web-based data collection occurring every two months (T1 to T9). Data collection between T9 and T10 occurred six months apart followed by T11, which occurred six months after T10. Final data collection took place in August 2022 (T11). Participant response rates over the survey time frame varied from 72.5% and 60.7–73.9%. This study follows the Strengthening the Reporting of Observational Studies in Epidemiology (STROBE) guidelines [49].

### Timeline of the COVID-19 pandemic in Ontario

Data collection took place during various COVID-19 waves, public health measures, and pandemic relief programs [27, 50–53]. Figure 2 provides a timeline of the survey time points, COVID-19 waves, public health measures and the CERB.

### Survey Procedure

Participants received an email containing a unique link to an online survey using REDCap electronic software [55]. Participants gave informed consent through REDCap and filled out self-report questionnaires about their demographics, mental health, substance use, health and social behaviour, and lifestyle changes. For each time point, the survey was available for a minimum of three weeks. Reminder emails to complete the survey were sent to participants every 2–3 days. Participants received a $15-$35 gift card as honorarium at each wave.

### Measures

*NEET*. The survey asked participants whether they were currently a student (full time; part-time; or not in school); and their current employment status (full-time, part-time, self-employed, volunteering, training/apprenticeship, or unemployed). We classified participants as NEET as youth who were not in school (not in part- or full-time formal education), were unemployed (not in paid or temporarily absent from work at the time of the survey) and inactive, and not in a training programme following the OECD definition [56].

*Mental Health and Substance Use Measures.* We used the CoRonavIruS Health Impact Survey (CRISIS) [57], Youth Self-Report Baseline V.0.1., developed by the National Institute of Mental Health in the USA. This survey consists of six subscales with varying items per subscale. For the second wave, we updated the CRISIS survey to include V.0.3. items. Key differences between V.0.1 and V.0.3 were items add to the daily behaviours; emotions/worries; and media use subscales. We used the Mood States subscale of the CRISIS, which includes 10 items, measuring mood and anxiety (sadness, enjoyment, irritability and concentration issues, among others) during the past two weeks [58]. Participants responded using a five-point Likert scale (scored from 0 to 4). As the psychometric properties for the Youth Self-Report version have not been published yet [59], we followed Nikolaidis et al. (2021) and scores were summed and a mean score was calculated for the scale to describe pooled results [60]. Mood States scale sum scores were analyzed as a continuous measure, with higher scores representing more difficulties across the study timeframe.

The Global Appraisal of Individual Needs Short Screener (GAIN-SS) (version 3) [61] was also used in the study to screen for (i) internalizing disorders (depression, anxiety, trauma, somatic complaints etc.); (ii) externalizing disorders (impulsivity, conduct problems, hyperactivity, attention deficits etc.) and (iii) substance use disorders across the study timeframe. The GAIN-SS also includes a crime/violence screener, however, low rates of endorsement precluded its inclusion within the current study. Participants indicated how recently they had experienced significant difficulty with each symptom “never” to “within the past month”. Symptoms endorsed in the past month were counted and summed within each domain subscale, and scores could range from 0 to 6 for the Internalizing domain, 0–7 for the Externalizing domain, and 0–5 for the Substance Use Problems domain. Existing evidence regarding the GAIN-SS suggests that three or more items endorsed within the past month indicate a high likelihood of meeting diagnostic criteria and/or a need for services within that domain [61, 62]. We assessed normality of each domain subscale score through histograms. The internalizing and externalizing scores were analyzed as ordinal measures and categorized as high (3 + endorsements within the past month); moderate (1–2 endorsements within the past month) and no symptoms (0 endorsements within the past month). Given that the substance use disorder domain scores were positively skewed even after log transformation, we dichotomized the scores: “no substance use problems in the past month” and “any substance use problems in the past month”.

The PTSD Checklist for DSM-5 (PCL-5) [63] is a 20-item scale used to measure the presence and severity of symptoms of PTSD, based on the Diagnostic and Statistical Manual of Mental Disorders, fifth edition (DSM-5) diagnostic criteria. The PCL-5 was adapted to the COVID-19 pandemic by the study team, wherein participants were asked about PTSD symptoms specifically related to stressful life experiences related to the COVID-19 pandemic and rated how bothered they were by these experiences on a five-point Likert scale from “not at all” (scored as 1) to “extremely” (scored as 5). Data on the PCL-5 were collected from the T2 time point onwards. Items were summed to provide a total severity score. The PCL-5 was analyzed as a log-transformed continuous measure across the study timeframe, due to the skewed distribution.

*Covariates*. Demographic characteristics were collected at T1. We included age (continuous measure); gender identity (man/boy [cis]; woman/girl [cis]; Transgender, non-binary or gender diverse); ethnicity (White; Black; East Asian; South Asian; and another background); immigrant status (born in or outside of Canada); living arrangement (own apartment/home; with parents or family; friends/peers; or precarious housing); and area of residence (large city and suburbs of large city; small city, town, village or rural area).

### Statistical analysis

Statistical analyses were performed using SAS Enterprise Guide version 7.1 (SAS Institute Inc., Cary, NC, USA) [64].

Descriptive summaries were calculated for all study variables overall and by NEET status. Cronbach’s alpha (α) was used to assess internal consistency of the scales and subscales. Chi-squared tests (χ^2^) were used to compare NEET status at T1 vs. study participation rates at T11. Study participation was defined as having non-missing NEET status at T11. Chi-squared, Fisher’s Exact, and Cramer’s V tests were used to analyze NEET status, MHSU measures, and demographic variables at T1 with T11 study participation. To model the trajectory of MHSU conditions between NEET status over time, linear mixed effects models were used to model associations of NEET status, time, and NEET-by-time interactions on CRISIS (Mood States) scores across 11 time points and PCL-5 scores across 10 time points, adjusting for covariates and the random effects of participants. Generalized estimating equations (GEE) were used to analyze associations of NEET status, time, NEET-by-time interactions on the ordinal (internalizing and externalizing) and binary (substance use problems) GAIN-SS scores across 11 time points, adjusting for covariates. To have the most flexibility and capture changes across the course of the study, time was treated as categorical. Time was not viewed as a linear trend. Given that the study was over a long time period (11 time points over 2 years), with many events taking place over the course of the pandemic, we did not expect time to have a linear effect. Pairwise contrasts of MHSU problems were estimated per time point between NEET and non-NEET youth. Odds ratios, estimated marginal means, and their 95% confidence intervals (CI) were reported. Forward model selection was used to add significant covariates (p < 0.05) to each model. Diagnostic plots were used to assess model assumptions. Given the exploratory nature of this study, no formal adjustments or multiple comparisons were applied. Findings from this study would need to be validated in an independent sample.

As an exploratory analysis, we examined whether covariates such as age, gender identity, ethnicity and area of residence modified the association between NEET status, the interaction between NEET status and time, and each MHSU measure by investigating two- and three-way interactions. For two-way interactions, these models included the main effect of NEET status, the main effect of time, the interaction of time and NEET status, the main effects of the covariates, and covariate interactions with NEET status as fixed effects. For three-way interactions, models included the main effect of NEET status, the main effect of time, the interaction of time and NEET status, the main effects of the covariates, and covariate interactions with NEET status and time as fixed effects.

### Missing data

For each outcome scale, participant observations missing > 50% items were excluded from analysis; mean imputation was used for observations missing ≤ 50% items. Missingness patterns per outcome were summarized per time point. Linear mixed and GEE models included all non-missing participant observations. These methods are generally robust to missing data [65].

## Results

Table 1 illustrates the baseline (T1) characteristics of participants overall and by NEET status. NEET youth represented 18.9% of the sample at T1 (n = 117). The mean age for NEET youth was 21.6 years (range 17–27 years). Across the time points, from 45 to 49% of youth reported their income reduced. Missing data patterns for NEET status and outcome measures are reported in Additional File 1.

**Table 1:** Demographic characteristics of participants at T1 (n = 618)

	NEET at T1 (n = 117)		non-NEET at T1 (n = 496)		Overall (n = 618)	
	Mean	Range	Mean	Range	Mean	Range
**Age**	21.6	17–27	20.3	14–28	20.6	14–28
	**n**	**%**	**n**	**%**	**n**	**%**
**Gender**						
Man/boy	38	32.5	162	32.7	202	32.7
Woman/girl	71	60.7	313	63.1	387	62.6
Transgender, non-binary, gender diverse	8	6.8	21	4.2	29	4.7
**Ethnicity**						
Asian (East)	5	4.3	34	6.8	40	6.5
Asian (South)	8	6.8	63	12.7	71	11.5
Black	2	1.7	25	5.0	28	4.6
White	70	59.8	305	61.5	377	61.2
Another ethnicity	32	27.3	69	13.9	100	16.2
IBPOC (for analysis only)	47	40.2	191	38.5	239	38.8
**Born in Canada**						
Yes	105	89.7	433	87.3	541	87.7
No	12	10.3	63	12.7	76	12.3
**Living arrangement**						
Own apartment/home	20	17.2	61	12.3	82	13.3
With parents or other family members	70	60.3	372	75.0	445	72.1
With friends/peers	21	18.1	44	8.9	65	10.5
Precarious housing	5	4.3	19	3.8	25	4.0
**Area of residence**						
City (large, suburbs)	79	67.5	330	66.5	413	66.8
Small city, town or village, rural area	38	32.5	166	33.5	205	33.2
**GAIN-SS**						
Internalizing disorder*	76	66.7	242	50.0	318	53.2
Externalizing disorder*	28	24.6	137	28.3	165	27.6
Substance use problems	18	15.8	53	10.9	71	11.8

*The proportion of participants at an increased likelihood of currently meeting diagnostic criteria or a need for services using the GAIN Short Screener. NEET: not in employment, education, or training; IBPOC: Indigenous, Black, and People of Colour

Analyzing the GAIN-SS sub-scales at T1, NEET youth were significantly more likely to screen positive for an internalizing disorder compared to non-NEET youth (NEET 66.7% vs. non-NEET 50%) (OR = 1.92; 95%CI=[1.26–2.91] *p* = 0.002). Similarly, 46.5% of NEET youth and 37.3% of non-NEET youth met threshold for substance use problems, although these differences were not significant (OR = 1.46; 95%CI=[0.97–2.20] *p* = 0.07). Meanwhile, 24.6% of NEET youth and 28.3% of non-NEET youth met diagnostic criteria for an externalizing disorder; these differences were not significant (OR = 0.82; 95%CI=[0.57–1.18] *p* = 0.29). At the follow-up time points, NEET youth represented 11.6% of the sample at T2, 11.5% at T3, 8.8% at T4, 7.7% at T5, 10.9% at T6, 9% at T7, 11.4% at T8, 9.6% at T9, 7.7% at T10, and 10.8% at T11. NEET youth (47%) vs. non-NEET youth (31.7%) at baseline were significantly less likely to participate at T11 (χ^2^ [1] = 9.87, *p* = 0.002). At T1, there were no significant differences in MHSU measures between NEET youth and T11 participation. There was a significant difference found between T1 gender identity and T11 participation rates (χ^2^ [2] = 6.93, *p* = 0.03); 60.5% (n = 23) of NEET youth who identify as boys/men dropped out of the study at T11.

Cronbach’s α for the following indices were: GAIN-SS subscale for internalizing disorder (α = 0.90), subscale for externalizing disorder (α = 0.89), and subscale for substance use disorder (α = 0.89); PCL-5 (α = 0.91); and CRISIS Mood States (α = 0.90).

### CRISIS

We separately examined the effects of selected baseline covariates on Mood States scores over time. Younger youth (14–17 years) had significantly higher mean Mood State scores compared to older youth (18–28 years) across all time points (mean difference per year decrease [d] = 0.02, CI: 0.004–0.03). Youth who identify as Trans, non-binary or gender diverse had significantly higher mean Mood State scores compared to youth who identify as cisgender boys/men (d = 0.58, 95%CI=[0.39–0.77]) or girls/women (d = 0.29, 95%CI=[0.11–0.48]) across all time points, indicating worse mental health. Youth who identify as White had significantly higher mean Mood State scores compared to those from another background (d = 0.12, 95%CI=[0.04–0.19]) across all time points. Youth who lived in large cities and suburbs of large cities had significantly higher mean Mood State scores compared to those in small cities, towns, villages and rural areas (d = 0.14, 95%CI[0.06–0.22]).

Overall, there were no significant differences in mean Mood State scores (NEET-by-time interaction *p* = 0.47) between NEET youth and non-NEET youth across the 11 time points, adjusting for age, gender identity, ethnicity and area of residence (Table 2). Pairwise contrasts showed that NEET youth tended to have lower mean Mood State scores compared to non-NEET youth only at T7 (d=-0.13; 95%CI=[-0.26- -0.003]; *p* = 0.04) (Table 2). Two- and three way interactions showed that age, gender identity, ethnicity and area of residence did not modify the effect of NEET youth or NEET status interacting with time on mean Mood State scores (data not shown).

**Table 2:** Estimated pairwise differences in CRISIS and PCL-5 scores, NEET vs. non-NEET status across time ^a^

	Final covariates	T1 (n = 117)	T2 (n = 52)	T3 (n = 47)	T4 (n = 36)	T5 (n = 31)	T6 (n = 42)	T7 (n = 40)	T8 (n = 48)	T9 (n = 36)	T10 (n = 35)	T11 (n = 44)	*p*
CRISIS	Age, gender identity, ethnicity, area of residence	0.07 (-0.01-0.16)	-0.03 (-0.15-0.08)	0.00 (-0.12-0.13)	-0.04 (-0.18-0.10)	0.01 (-0.14-0.15)	0.04 (-0.08-0.17)	-0.13^b^ (-0.26-0.00)	-0.02 (-0.13-0.10)	0.07 (-0.07-0.20)	0.03 (-0.11-0.17)	-0.001 (-0.12-0.12)	0.47
Log(PCL-5)	Age, gender identity, and area of residence	Not collected at T1	-0.03 (-0.11-0.05)	-0.01 (-0.10-0.07)	-0.06 (-0.16-0.03)	-0.03 (-0.13-0.07)	0.07 (-0.02-0.15)	0.01 (-0.02-0.15)	0.05 (-0.03-0.13)	0.03 (-0.06-0.12)	0.09 (0.00-0.18)	0.11^b^ (0.03–0.19)	0.09

^a^ Positive values indicate higher outcome scores in NEET vs. non-NEET youth

^b^ Significant time points (*p* < 0.05)

### GAIN-SS

*Internalizing Disorder.* We separately examined the effects of selected baseline covariates on Internalizing disorder scores over time. Younger youth (14–17 years) had significantly higher odds of screening positive for an internalizing disorder compared to older youth (OR = 1.06, 95%CI=[1.00-1.11]) across all time points. Youth who identify as Trans, non-binary or gender diverse had significantly higher odds of screening positive for an internalizing disorder compared to youth who identify as cisgender boys/men (OR = 8.33, 95%CI=[4.17–16.67]) and girls/women (OR = 3.70, 95%CI=[1.96–7.14]) across all time points. Youth living in cities and suburbs of large cities had significantly higher odds of screening positive for an internalizing disorder compared to those in small cities, towns, villages and rural areas (OR = 1.35, 95%CI=[1.03–1.76]) across all time points.

Overall there were no significant differences in the odds of screening positive for an internalizing disorder (NEET-by-time interaction *p* = 0.33) between NEET youth and non-NEET youth across the 11 time points, adjusting for age, gender identity and area of residence (Table 3). Pairwise contrasts showed that NEET youth had higher odds of screening positive for an internalizing disorder compared to non-NEET youth at T1 (OR = 2.49, 95%CI=[1.14–5.40], *p* = 0.02), T9 (OR = 2.10, 95%CI=[1.37–3.22], *p* = 0.001) and T10 (OR = 1.98, 95%CI=[1.07–3.66], *p* = 0.03). Indeed, NEET youth had twice the odds of screening positive for internalizing disorders compared to non-NEET youth across these specific time points (Table 3). Two- and three way interactions showed that age, gender identity or area of residence did not modify the effect of NEET status or NEET status interacting with time on internalizing disorders (data not shown).

**Table 3:** Estimated Odds Ratios in GAIN-SS scores between youth with NEET vs. non-NEET status across time ^a^

	Final covariates	T1 (n = 117)	T2 (n = 52)	T3 (n = 47)	T4 (n = 36)	T5 (n = 31)	T6 (n = 42)	T7 (n = 40)	T8 (n = 48)	T9 (n = 36)	T10 (n = 35)	T11 (n = 44)	*p*
Internalizing disorder	Age, gender identity, and area of residence	2.49 ^b^ (1.15–5.40)	1.36 (0.74–2.47)	1.16 (0.61–2.23)	0.74 (0.36–1.51)	1.60 (0.84–3.02)	1.00 (0.52–1.95)	1.51 (0.81–2.81)	1.92 (0.97–3.80)	2.10^b^ (1.37–3.22)	1.98^b^ (1.07–3.66)	1.12 (0.63–2.01)	0.33
Externalizing disorder	Age, gender identity, and area of residence	0.77 (0.53–1.11)	0.68 (0.41–1.14)	1.09 (0.60-2.00)	0.52 (0.27–1.01)	0.79 (0.40–1.54)	0.74 (0.39–1.39)	0.67 (0.36–1.24)	0.82 (0.46–1.46)	1.56 (0.77–3.19)	1.20 (0.61–2.34)	1.47 (0.82–2.61)	0.37
Substance use problems	Age and ethnicity	1.04 (0.49–2.21)	1.29 (0.70–2.40)	1.52 (0.73–3.15)	0.80 (0.35–1.84)	0.86 (0.42–1.78)	0.87 (0.41–1.82)	0.93 (0.49–1.77)	0.97 (0.45–2.09)	1.32 (0.86–2.02)	0.99 (0.50–1.94)	0.57 (0.29–1.09)	0.43

^a^ Odds ratios > 1 indicate higher odds of having the disorder in NEET vs. non-NEET youth

^b^ Significant time points (*p* < 0.05)

*Externalizing Disorder*. We separately examined the effects of selected baseline covariates on Externalizing disorders scores over time. Youth who identify as Trans, non-binary or gender diverse had significantly higher odds of screening positive for externalizing disorders compared to youth who identify as cisgender boys/men (OR = 3.23, 95%CI=[1.85–5.56]) and girls/women (OR = 3.70, 95%CI=[1.28–3.70]) across all time points. Youth living in cities and suburbs of large cities had significantly higher odds of screening positive for externalizing disorders compared to those in small cities, towns, villages and rural areas (OR = 1.7, 95%CI=[1.31–2.20]) across all time points.

There were no significant differences in the odds of screening positive for an externalizing disorder (NEET-by-time interaction *p* = 0.37) between NEET youth and non-NEET youth across the 11 time points, adjusting for age, gender identity and area of residence. Pairwise contrasts showed no significant differences in externalizing disorders between time point and NEET status (Table 3). Two- and three way interactions showed that gender identity and area of residence did not modify the effect of NEET status or NEET status interacting with time on externalizing disorders.

*Substance Use Problems*. We separately examined the effects of selected baseline covariates on Substance Use domain scores over time. Older youth (20–28 years) had significantly higher odds of screening positive for substance use problems compared to younger youth (OR = 1.11, 95%CI=[1.04–1.18]) across all time points. Meanwhile, youth who identify as White had significantly higher odds of screening positive for a substance use problems compared to those who identify as IBPOC (South Asian, East Asian, Black, and another) (OR = 2.38, 95%CI=[1.72–3.23]).

There were no significant differences in the odds of screening positive for substance use problems (NEET-by-time interaction *p* = 0.43) between NEET youth and non-NEET youth across the 11 time points, adjusting for age and ethnicity. Pairwise contrasts showed no significant differences in substance use problems between time point and NEET status, adjusting for age and ethnicity (Table 3). Two- and three way interactions showed that age, gender identity or area of residence did not modify the effect of NEET status or NEET status interacting with time on substance use problems.

### PCL-5

We separately examined the effects of selected baseline covariates on PCL-5 scores over time. Younger youth (14–17 years) had significantly higher mean log PCL-5 scores compared to older youth (mean difference [d] = 0.02, 95%CI=[0.002–0.03]) across all time points. Youth who identify as Trans, non-binary or gender diverse had significantly higher mean log PCL-5 scores compared to youth who identify as cisgender boys/men (d = 0.35, 95%CI=[0.19–0.51]) and girls/women (d = 0.21, 95%CI=[0.05–0.37]) across all time points. Youth living in cities and suburbs of large cities had significantly higher mean log PCL-5 scores compared to those in small cities, towns, villages and rural areas (d = 0.14, 95%CI=[0.08–0.21]) across the 10 time points.

There were no significant differences in mean log PCL-5 scores (NEET-by-time interaction *p* = 0.09) between NEET youth and non-NEET youth across the 10 time points, adjusting for age, gender identity and area of residence. Pairwise contrasts showed that NEET youth tended to have higher mean log PCL-5 scores compared to non-NEET youth at T11 (d = 0.11, 95%CI=[0.03–0.19], *p* = 0.01), adjusting for age, gender identity and area of residence (Table 2). Two- and three way interactions showed that age, gender identity or area of residence did not modify the effect of NEET status or NEET status interacting with time on PTSD symptoms.

## Discussion

The current study investigated the MHSU of youth in Canada with NEET status longitudinally over the COVID-19 pandemic (April 2020–August 2022). Results indicate that at the start of the pandemic (April 2020, T1), a greater proportion of NEET youth had MHSU problems compared to non-NEET youth. With the exception of a few study time points, the differences in MHSU concerns between NEET youth and non-NEET youth were no longer significant after T1. Indeed, results indicate that several sociodemographic factors, including age, gender identity, ethnicity and area of residence influenced MHSU over the course of study time period during the pandemic. Although there were some study time points where NEET status was correlated with poor youth mental health, overall NEET status was not consistently associated with MHSU over and above these sociodemographic factors across the study. Similar results were found in a longitudinal cohort study in the UK [66].

Differences in MHSU problems were observed among youth subgroups over the study time period. Younger youth had greater mental health problems compared to older youth. Prior research has been mixed on the association between age and mental health problems during the COVID-19 pandemic, with some studies showing mental health symptoms elevated among older compared to younger youth [67, 68]. Greater mental health concerns among younger youth could potentially be associated with public health measures enacted during COVID-19, including school closures and stay-at-home orders [69]. These measures were unique to jurisdictions and could contribute to variations in mental health concerns. In addition and in line with prior research during the COVID-19 pandemic [70, 71], youth who identify as Trans, non-binary or gender diverse had greater mental health problems compared to youth who identify as cisgender boys/men and girls/women. It should be noted that our study had very low participation of youth who identify as Trans, nonbinary or gender diverse which could have influenced the findings. Previous research has showed that other factors that could contribute to greater mental health problems among Trans, non-binary or gender diverse youth include isolation from supportive peers and communities; living with unsupportive families and not feeling safe at home; and disruption to health and social services [70–73]. Future research should explore the impact of these factors on the MHSU of youth who identify as Trans, non-binary or gender diverse. Aligning with previous research older youth were more likely to screen positive for substance use problems which could be attributed to the greater exposure and duration of substance use [74, 75]. We also found that White youth screened positive for substance use problems compared to youth of another background. This finding is consistent with some research during the pandemic [76] and could be influenced by multiple, interacting factors related to family (e.g., parenting style), friends (e.g., peer pressure), and the community (e.g., promoting drinking behaviours) [77–80]. Further research, however, is needed on racial and ethnic differences in substance use among youth during the pandemic. Our findings also indicate that mental health problems were higher among youth living in urban compared to rural areas. Research prior to and during the pandemic have shown similar findings [81, 82] and could be attributed to population density and the related heightened impacts of public health measures on urban living, along with differential experiences of employment impacts, noise, and pollution. Further research is needed to explore the underlying mechanisms associated with these differences.

Our findings indicate that there were no differences in MHSU problems between NEET youth and non-NEET youth across the study timeframe. The finding could be attributed to several potential factors. Firstly, school and work can play a protective role on youth mental health helping youth achieve developmental milestones [83–85]. Indeed, education and jobs provide youth the opportunity for routine and structure, social support and feelings of belongingness, productivity, and the ability to develop different and new roles [71, 84, 86]. During the pandemic, non-NEET youth lost connection to school and work, which could be associated with adverse mental health symptoms [84], potentially contributing to no differences in MHSU problems between NEET and non-NEET youth. Youth in school experienced rapid and evolving changes to school structure, support, teaching and learning. There was also inconsistent information and expectations around student workload and the delivery of synchronous and asynchronous teaching [84, 87, 88]. These changes varied by classroom, schools and jurisdictions creating uneven learning, confusion, and stress for youth and families [84], potentially contributing to our finding that there were no differences in MHSU concerns between NEET and non-NEET youth. Moreover, there remained gaps in remote learning with 12% of Ontarians reporting unstable internet access and 65-75% of low-income houses having a home computer in 2020, creating learning losses and stress [84, 89–91]. Further, youth apprenticeships and co-op programs were cancelled or suspended, making the transition to the labour market more challenging for youth [84, 92]. For youth who worked during the pandemic, they were exposed to reduced hours, job insecurity, low wages, greater job demand, long periods of quarantine or isolation, low social support, uncertainty about the future, and potential employee rights violations [93], potentially contributing to our finding that there were no differences in MHSU concerns between NEET and non-NEET youth.

At the same time, pandemic relief programs and public health measures implemented in Ontario may have played a protective role on the MHSU of NEET youth across the study timeframe. The majority (88.1%) of youth 20–24 years of age received CERB benefits and the GST/HST top-up in 2020 [26]. Future research on how (if at all) pandemic relief programs and public health interventions influenced youth MHSU is warranted. In addition, the majority of NEET youth reported living with family or friends, which could have enhanced their perceived support and played a protective role on their MHSU. Indeed, social support from family, friends, and romantic partners has been shown to be critical for youth mental health and development [94, 95]. Findings from a recent systematic review on the impact of COVID-19 on adolescent mental health showed that a lack of social support during the COVID-19 pandemic was associated with increased levels of anxiety and depression in youth [96, 97]. Further research should investigate the association between the MHSU of NEET youth and social support during the COVID-19 pandemic.

Our findings showed that there were significantly less NEET youth participating in the study by T11. Results showed that over 60% of NEET youth who identify as boys/men had left the study by T11. A systematic review and meta-analysis on longitudinal cohort studies showed similar results [98]. Reasons for non-participation rates among boys/men could be attributed to time constraints and lack of interest to complete the survey over several time periods [99]. Future research could investigate what retention strategies would work among youth who identify as boys/men in longitudinal cohort studies.

Our study was constrained by some of the mental health measures used. We expected the CRISIS Mood States scale to yield similar mental health results as the GAIN-SS Internalizing Disorder sub-screener, however, NEET youth did not report greater mental health concerns on the Mood States scale compared to non-NEET youth. To identify the presence or absence of a MHSU problem and better respond to youth MHSU needs, measuring the MHSU outcomes of youth requires measures to be valid and reliable [100]. The reliability and validity of the GAIN-SS [62], including the Internalizing Disorder sub-screener [100], have been established. However, the psychometric properties of the CRISIS Mood States scale has not yet been published within a youth population, potentially influencing the study findings and necessitating future research in this area [59, 101]. In addition, many youth who participated in the current study were recruited from service-seeking studies. Prior evidence has showed that NEET youth from service-seeking studies reported fewer MHSU problems [8, 11, 16] compared to those in the general population [19, 102–105]. As such, recruiting from these groups could have biased our results and limited the generalizability of the findings. The study was also constrained by the non-representativeness of the sample and is a limitation of this study. Furthermore, we were unable to assess the duration of NEET status due to drop-out rates and missingness of data. Not being able to assess this duration limited our ability to investigate the proportion of youth who were temporarily disengaged or permanently inactive, and its effects on youth MHSU. In addition, we did not have data on these variables prior to the onset of the pandemic, so it is difficult to determine how these results compare to pre-pandemic times.

In this exploratory study, although we found significant differences in mental health problems between NEET and non-NEET youth at certain study time points, we cannot definitively say whether the differences were attributed to events related to the pandemic (e.g., COVID waves, lockdowns etc.), or social and individual risk factors (e.g., domestic abuse, household insecurity), as it was beyond the scope of the study. Factors that could be implicated with these findings are feelings of isolation at specific time points [38, 106], financial strain [107], trauma associated with losing family members and friends [108, 109], health-related fears [110, 111], and exposure to household conflict and abuse [112, 113]. This was beyond the remit of the current study. Further research on the social and individual risk factors at these time points could help elucidate the underlying mechanisms driving MHSU problems among NEET youth. At the same time, the current study did not measure socioeconomic status (SES). It would be important for future studies to investigate SES, MHSU and NEET status, as prior evidence has showed that youth from low SES are at greater risk of developing a mental health problem compared to their high SES counterparts [114–117]. These youth are exposed to a greater frequency of factors that contribute to stress (e.g., parental stress, limited-resource neighbourhoods) and mental health problems during this stage of life. Indeed, and in addition, the family and home environment are important factors to consider in NEET youth MHSU. These factors were not measured in the current study but would be important to investigate in future studies as a recent scoping review showed that family MHSU, composition, and health issues, as well as poor family support are associated with NEET status [118]. We also did not investigate pre-existing mental health conditions and NEET status, as this was beyond the scope of the current study. Future studies should conduct research on the bidirectional relationship between MHSU and NEET status.

Furthermore, our findings did not indicate that age, gender identity, ethnicity and area of residence moderated the association between NEET status and MHSU problems. This finding is consistent with prior research [12, 15]. However, further research on differences in MHSU and NEET subgroups should be conducted, as this research would help inform interventions and programs for youth at-risk of becoming NEET as well as the prevention of MHSU among NEET youth [15].

The findings from the study illustrate an opportunity to develop interventions and programs for specific subgroups, particularly those living in urban settings. Building on lessons learned and the adverse MHSU outcomes associated with the COVID-19 pandemic, it would be important to prepare for future pandemics. One way that this could be achieved is by strengthening integrated youth services to meet the needs of these youth groups [119]. Ontario has dedicated community-based integrated youth services that provide developmentally appropriate and holistic care to young people 12–25 years. These services include MHSU, physical health, educational, housing supports, peer support, and navigation support and are known as Youth Wellness Hubs Ontario (YWHO). Strengthening YWHO services could be an important approach since prevention and early intervention have been shown to mitigate MHSU problems [120]. In addition, services could engage diverse youth in various settings to ensure that services, including treatment protocols, are adapted to their needs. Services should also consider training health care professionals on providing gender-affirming care. As well, given the increase in virtual services during the pandemic [121, 122], strengthening the delivery of safe and supportive virtual platforms could increase access and utilization of these services.

## Conclusions

The exploratory study contributes to the limited evidence on associations between the COVID-19 pandemic and the MHSU of Canadian NEET and non-NEET youth. Our findings indicate that sociodemographic factors such as age, gender identity, ethnicity and area of residence were associated with youth MHSU over the course of the study and during the pandemic. We did not find differences in MHSU problems between NEET and non-NEET youth across the timeframe. Our analysis offers a starting point for future in-depth quantitative and qualitative studies among this population group, particularly among service seeking youth. It also adds to the evidence on NEET status and MHSU problems among youth longitudinally.

### Electronic supplementary material

Below is the link to the electronic supplementary material.

**Additional File 1: Additional Table 1.** Missing data patterns for NEET status and outcome measures over 11 time points.

## Data Availability

Data are available upon reasonable request. Data are available upon Research Ethics Board approval.

## Abbreviations

CAMH: Centre for Addiction and Mental Health
CERB: Canada Emergency Response Benefit
CI: Confidence intervals
COVID-19: Coronavirus disease 2019
CRISIS: CoRonavIruS Health Impact Survey
DSM: Diagnostic and Statistical Manual of Mental Disorders
GAIN-SS: Global Appraisal of Individual Needs Short Screener
GEE: Generalized estimating equations
GST/HST: Goods and Services Tax/Harmonized Sales Tax
MHSU: Mental health and substance use
NEET: Not in education, employment, or training
PCL-5: PTSD-Checklist for DSM-5
OECD: Organisation for Economic Co-operation and Development
PTSD: Post-traumatic stress disorder
STROBE: The Strengthening the Reporting of Observational Studies in Epidemiology (STROBE)
YWHO: Youth Wellness Hubs Ontario
