# Supplementary material for: Associations over the COVID-19 pandemic period and the mental health and substance use of youth not in employment, education or training in Ontario, Canada: a longitudinal, cohort study
Source: Child Adolesc Psychiatry Ment Health. 2023 Sep 7;17:105. doi: 10.1186/s13034-023-00653-4 (PMC10486040; doi:10.1186/s13034-023-00653-4)
Supplement: Supplementary file 1 — Additional File 1: Additional Table 1. Missing data patterns for NEET status and outcome measures over 11 time points. [file 13034_2023_653_MOESM1_ESM.pdf]

Additional Table 1. Missing data patterns for NEET status and outcome measures over 11 time points

Variable		T1	T2	T3	T4	T5	T6	T7	T8	T9	T10	T11
NEET status	N	613	448	404	406	402	384	441	417	372	455	404
	Nmiss	5	0	4	5	0	1	2	3	3	2	3
GAIN-SS internalizing	N	603	440	403	406	397	382	437	412	362	456	400
	Nmiss	15	8	5	5	5	3	6	8	13	1	7
GAIN-SS externalizing	N	603	440	402	405	397	382	437	411	362	455	399
	Nmiss	15	8	6	6	5	3	6	9	13	2	8
GAIN-SS substance use	N	604	440	403	404	396	381	436	411	362	455	399
	Nmiss	14	8	5	7	6	4	7	9	13	2	8
PCL-5	N	-	433	398	406	395	378	435	410	361	455	401
	Nmiss	-	15	10	5	7	7	8	10	12	2	6
CRISIS	N	616	448	407	411	402	385	443	419	375	456	405
	Nmiss	2	0	1	0	0	0	0	1	0	1	2
